# ﻿Karyotype characteristics and gene COI sequences of *Chironomusbonus* Shilova et Dzhvarsheishvili, 1974 (Diptera, Chironomidae) from the South Caucasus (Republic of Georgia, Paravani river)

**DOI:** 10.3897/CompCytogen.v16.i1.79182

**Published:** 2022-03-01

**Authors:** Mukhamed Kh. Karmokov

**Affiliations:** 1 Tembotov Institute of Ecology of Mountain territories RAS, I. Armand str., 37a, Nalchik 360051, Russia Tembotov Institute of Ecology of Mountain territories RAS Nalchik Russia

**Keywords:** The study presents data on the karyotype characteristics and the mitochondrial gene COI sequences of the non-biting midge *Chironomusbonus* Shilova et sDzhvarsheishvili, 1974 (Diptera, Chironomidae) from the South Caucasus. The species belongs to the *Ch.plumosus* group of sibling species, one of the most widespread and successful groups in the genus *Chironomus* Meigen, 1803. The karyotype of the studied population is monomorphic. The morphological and chromosomal characteristics of *Ch.bonus* from the Caucasus are similar to those previously described for this species (Kiknadze et al. 1991a). In the phylogenetic tree based on the COI gene sequences, one can observe several clear clusters. We named them Palearctic *Ch.plumosus*, Far Eastern *Ch.borokensis*-*Ch.suwai*, and Nearctic *Ch.entis*-*Ch.plumosus* clusters. The calculated K2P genetic distances within each cluster have not exceeded the 3% threshold for the genus *Chironomus*. Contrary to this, the distances between the clusters exceed this range and correspond to separate species. The *Ch.bonus* sequences belong to the cluster consisting of *Ch.plumosus* (Linnaeus, 1758) sequences from European populations, and do not form a separate clade of the phylogenetic tree. One can suppose that the origin of the *Ch.plumosus* group of sibling species dates back to 5.75–3.43 million years ago (Mya), the epochs of Late Miocene (7, 3–5, 3 Mya) and early Pliocene (5, 3–2, 58 Mya). On the other hand, Palearctic *Ch.plumosus*, Far Eastern *Ch.borokensis*-*Ch.suwai*, and Nearctic *Ch.entis*-*Ch.plumosus* clusters appeared relatively recently in the Middle Pleistocene, 1.288–0.307 Mya. The possible relationship between the climate changes in the Pliocene and the origin of the *Ch.plumosus* group are discussed. Chironomidae, *Chironomusbonus*, COI gene, Diptera, mitochondrial DNA, phylogeny, polytene chromosomes, South Caucasus

## ﻿Introduction

Shilova and Dzhvarsheishvili first described *Chironomusbonus* Shilova et Dzhvarsheishvili, 1974 from Paravani Lake in the Republic of Georgia (Shilova and Dzhvarsheishvili 1974). According to the Fauna Europaea web source (Pape and Beuk 2016), the species is known in Europe from the French mainland, Switzerland, and Bulgaria. The species has also been found in the Republic of Armenia (Sevan Lake) (Kiknadze et al. 2016).

The species *Ch.bonus* belongs to the *Ch.plumosus* group of sibling species, one of the most widespread and successful groups in the genus *Chironomus* Meigen, 1803. According to Shobanov (2000), the group of sibling species is a quasi-taxonomic category that unites species which are similar in morphology and karyotype. Often, there are no clear diagnostic criteria for groups of species, and the association is based on the principle of relative similarity. Shobanov (1989) and Kiknadze et al. (1991) developed the morphological characteristics of the *Ch.plumosus* group. These characteristics include several key features. In general, the larvae are relatively large, ranging from 16 to 30 mm. The larvae of most species belong to the *plumosus*-type, with the so-called sculpturing on the outer (ventral) side of the ventromental plates. Most species in the group prefer lowland rivers with slow current and high sediment silt. In addition, they are widely present in different types of ponds and lakes, of both natural and artificial origin. Several species of the group (at least *Ch.plumosus* (Linnaeus, 1758) and *Ch.borokensis* Kerkis, Filippova, Schobanov, Gunderina et Kiknadze, 1988, see below) can tolerate low oxygen concentrations for an extended period (Shobanov 2001).

According to Kiknadze et al. (2016), the group consists of 14 species: *Ch.agilis* Schobanov et Djomin, 1988; Chironomussp.propeagilis (syn. *Ch.agilis* 2) Kiknadze, Siirin et Filippova, 1991; *Ch.balatonicus* Dévai, Wülker et Scholl, 1983; *Ch.bonus* Shilova et Dzhvarsheishvili, 1974; *Ch.borokensis* Kerkis, Filippova, Shobanov, Gunderina et Kiknadze, 1988; *Ch.entis* Shobanov, 1989; *Ch.muratensis* Ryser, Scholl et Wülker, 1983; *Ch.nudiventris* Ryser, Scholl et Wülker, 1983; *Ch.plumosus*; *Ch.sinicus* Kiknadze, Wang, Istomina et Gunderina, 2005; *Chironomus* sp. J Kiknadze, 1991; *Chironomus* sp. K Golygina et Ueno, 2008; *Ch.suwai* Golygina et Martin, 2003; and *Ch.usenicus* Loginova et Belyanina, 1994. The identification of these species can only be done through karyological analysis (reviewed in Kiknadze et al. 1996; Butler et al. 1999). Most of them often occur sympatrically in the same body of water, which can severely complicate the identification process.

The majority of the species in the *Ch.plumosus* group have a Palearctic distribution. Only two of them, *Ch.plumosus* and *Ch.entis*, are also found in the Nearctic, and they can therefore be considered as Holarctic species (Butler et al. 1999; Kiknadze et al. 2000; Golygina and Kiknadze 2001). Adult morphology suggests that Palearctic *Ch.plumosus* has a very wide distribution range from Western Europe to the Far East (Linevich and Sokolova 1983). However, karyological analysis has shown that *Ch.borokensis* and Chironomussp.propeagilis replace *Ch.plumosus* in Eastern Siberia and the Far East (Kiknadze et al. 1996; Golygina et al. 2003). As indicated before, the karyotype study is the only reliable method for recognizing species in this group.

The karyotype of *Ch.bonus* has been described by Kerkis et al. (1989) and Kiknadze et al. (1991a). A short communication about the *Ch.bonus* karyotype was presented by Belyanina (1983). Some information on the karyotype and external morphology of *Ch.bonus* from Bulgaria was given by Michailova (1994). The biggest DNA databases, GenBank and BOLD, do not contain any DNA data on *Ch.bonus*, including sequences of the COI gene.

The aim of the work is to present the description of the karyotype and gene COI sequences of *Ch.bonus* from the South Caucasus, as well as to compare the karyotype characteristics and DNA data of *Ch.bonus* with the corresponding information available for other species of the *Ch.plumosus* group.

## ﻿Methods

**Figure 1.**
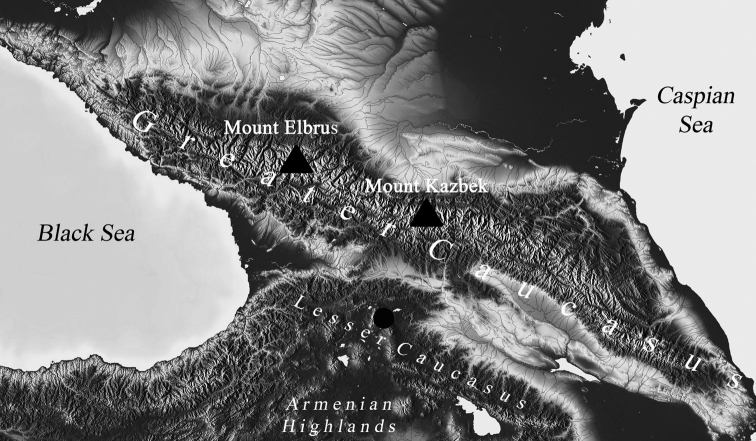
Collection site of *Ch.bonus* in South Caucasus. The collection site is marked with dark circle.

For both DNA and karyological studies, we used fourth-instar larvae of *Ch.bonus*. We collected larvae from a particular site in the Republic of Georgia (South Caucasus): 18.07.17, 41°19.305'N, 43°45.563'E, Ninotsminda district in the region of Samtskhe-Javakheti, one of the branches of the Paravani river, just 0.6 km north of Saghamo settlement, altitude of ca. 2000 m a.s.l. The maximum depth of the river is about 1 m, and the salinity of the water is about 40 ppm. The collection site is marked on the map with a dark circle (Fig. 1). The geographic division of the Caucasus follows Gvozdetsky (1963).

The head capsules and bodies of six larvae were slide-mounted in Faure-Berlese medium. The specimens have been deposited at the Tembotov Institute of Ecology of the Mountain Territories RAS in Nalchik, Russia. We studied the karyotype of all six larvae from the Caucasus region.

For karyological study, we fixed the larvae in an ethanol-glacial acetic acid solution (3:1). The preparations of the chromosomes were made using the ethanol-orcein technique (see Dyomin and Ilyinskaya 1988; Dyomin and Shobanov 1990). The banding sequences were designated as per the accepted convention, specifying the abbreviated name of the species, the symbol of the chromosome arm, and sequence number, as h’bonA1, h’bonB1, etc. (Keyl 1962; Wülker and Klötzli 1973).

We performed the identification of chromosome banding sequences for arms A, E, and F using photomaps by Kiknadze et al. (1991a, 2016) in the system of Keyl (1962) and chromosome mapping for arms C and D as per Kiknadze et al. (1991a, 2016) in the system of Dévai et al. (1989). The chromosome preparations were studied using a Carl Zeiss Axio Imager A2 microscope.

### ﻿DNA extraction, amplification and sequencing

We used four karyologically studied larvae of *Ch.bonus* for further DNA extraction. DNA was extracted from the larvae and preserved in 96% ethanol using a Diatom DNA Prep 100 kit (Izogen Laboratory Ltd, Moscow, Russia) according to the manufacturer’s protocol. DNA extraction was performed on vacuum-dried samples without prior homogenization. Samples were incubated in a lysis buffer at a temperature of 55.5 °C for 16 h. After the extraction, the head capsules were retrieved for dry mounting. The resulting DNA solutions were stored at -18 °C. The amplification of the mitochondrial COI gene was conducted using the MasterMix X5 kit (Dialat Ltd, Moscow).

To amplify the mitochondrial COI gene’s barcoding region, primers 911 (5´-TTTCTACAAATCATAAAGATATTGG-3´) and 912 (5´- TAAACTTCAGGGTGACCAAAAAATCA-3´) (Folmer et al. 1994) were used. PCR was performed in a 25-µL reaction volume. The amplification profile consisted of an initial step of 95 °C for 5 min, followed by 45 cycles of 95 °C for 30 s, 50 °C for 30 s, and 72 °C for 50 s, and finally an 8-min extension step at 72 °C, a final elongation at 72 °C (8 min), and final storage at 4 °C. The resulting PCR products were purified by precipitation in a 0.15 M CH_3_COONa solution in 90% ethanol and then rinsed with 70% ethanol. The results were visualized by 1.5% agarose gel electrophoresis with ethidium bromide.

Purified PCR products were sequenced in both directions. DNA sequencing of the COI gene was performed according to Sanger using the BigDye Terminator v3.1 commercial kit (ThermoFisher) and the ABI 3130×l genetic analyzer (ThermoFisher) at Syntol JSC (Moscow, Russia). The GenBank accession numbers of the three sequences obtained in this study are MZ014021, MZ014022, and MZ014023.

### ﻿Phylogenetic analysis

For the phylogenetic comparison, we used DNA data (sixty-one COI gene sequences) from both the GenBank and BOLD (Ratnasingham and Hebert 2007) databases. Accession numbers of used sequences in GenBank and BOLD: *Chironomusdorsalis* Meigen, 1818 (JN887047.1); *Ch.balatonicus* (JN016826.1); *Ch.muratensis* (AF192194.1); Chironomussp.propeagilis (AF192190.1); *Ch.borokensis* (AB740261.1); *Ch.usenicus* (JN016807.1, JN016809.1, JN016808.1); *Ch.entis* (KF278213.1, KF278212.1, KJ085531.1, KJ087284.1, KJ089893.1, GBDPC429-14, MGOCF102-16); *Ch.plumosus* (AB740263.1, AB740262.1, JN016830.1, JN016829.1, CHBAL014-20, CHIFI298-16, CHIFI299-16, LEFIJ3947-16, LEFIJ3948-16, PGBAL006-19, PGBAL007-19, PGBAL009-19, PGCBG089-20, BSCHI661-17, BSCHI063-11, BSCHI115-17, BSCHI219-17, BSCHI284-17, BSCHI350-17, BSCHI517-17, BSCHI644-17, GBDP44143-19, GBDP44180-19, LC050899.1, LC050900.1, JCDB364-15, JCDB363-15, GBDP11685-12, GBDP11686-12, GBDP11687-12, GBDP12282-12, XJDQD1039-18, XJDQD1037-18, XJDQD1038-18, XJDQD1036-18, MN750315.1, GBDPC430-14, SDP408034-15, GBDPC133-14, GBDPC138-14, GBDPC144-14, GBDPC166-14); and *Pagastiellaorophila* (Edwards, 1929) (JN265047.1).

We found some COI gene data in both the GenBank and BOLD databases only for seven species of the *Ch.plumosus* group out of 14. We used in our study COI gene sequences from both the aforementioned databases for *Ch.balatonicus*, *Ch.muratensis*, Chironomussp.propeagilis, *Ch.borokensis*, *Ch.usenicus*, *Ch.entis*, and *Ch.plumosus*, with available data for species with Holarctic and Nearctic distributions. The most abundant data on the COI gene are available for *Ch.plumosus* (GenBank and BOLD – 66 and 138 sequences, respectively) and *Ch.entis* (GenBank and BOLD – 339 and 13 sequences, respectively). DNA sequences of *Ch.plumosus* obtained from material collected from both Western and Eastern Europe, the Middle East, the Far East, and Northern America were included into the analysis. Concerning *Ch.entis*, available DNA sequences are more uniform and were obtained from material collected almost exclusively from Northern America (Canada). In cases when a large number of sequences were available from the same region, we used no more than 5–6 sequences with different haplotypes to avoid overloading the phylogenetic tree.

We conducted the alignment of COI sequences with MUSCLE with a genetic code of “invertebrate mitochondrial” packaged in MEGA 6 (Tamura et al. 2013). The pairwise sequence distances (Tables 1–4) consisting of the estimated number of base substitutions per site using MEGA 6 and the K2P model (Kimura 1980) were calculated. The analysis involved 61 nucleotide sequences. The codon positions included were 1^st^+2^nd^+3^rd^+Noncoding. All positions containing gaps and missing data were eliminated. There was a total of 579 positions in the final data set.

**Table 1.** Estimates of evolutionary divergence between sequences of Palearctic *Ch.plumosus* cluster. The number of base substitutions per site (%) from between sequences are shown. Analyses were conducted using the Kimura 2-parameter model (Kimura 1980).

№	Sequences	1	2	3	4	5	6	7	8	9	10	11	12	13	14	15	16	17	18	19	20	21	22	23	24	25
1	JN016807.1*Ch.usenicus* Russia, Saratov terr.	0																								
2	JN016809.1*Ch.usenicus* Russia, Saratov terr.	0.364	0																							
3	JN016808.1*Ch.usenicus* Russia, Saratov terr.	0.182	0.182	0																						
4	MZ014023*Ch.bonus* Georgia, S. Caucasus	0.547	0.547	0.364	0																					
5	MZ014022*Ch.bonus* Georgia, S. Caucasus	0.364	0.364	0.182	0.182	0																				
6	MZ014021*Ch.bonus* Georgia, S. Caucasus	0.547	0.547	0.364	0.364	0.182	0																			
7	JN016830.1*Ch.plumosus* Russia, Saratov Terr.	0.547	0.547	0.364	0.730	0.547	0.731	0																		
8	JN016829.1*Ch.plumosus* Russia Saratov Terr.	0.182	0.182	0	0.364	0.182	0.364	0.364	0																	
9	AB740263.1*Ch.plumosus* Russia	0.547	0.547	0.364	0.730	0.547	0.730	0.730	0.364	0																
10	AB740262.1*Ch.plumosus* Russia	0.182	0.182	0	0.364	0.182	0.364	0.364	0	0.364	0															
11	CHBAL014-20*Ch.plumosus* Montenegro	0.364	0.364	0.182	0.547	0.364	0.547	0.182	0.182	0.547	0.182	0														
12	PGBAL006-19*Ch.plumosus* Montenegro	0.364	0.364	0.182	0.547	0.364	0.547	0.182	0.182	0.547	0.182	0	0													
13	PGBAL007-19*Ch.plumosus* Montenegro	0.547	0.547	0.364	0.730	0.547	0.731	0.364	0.364	0.730	0.364	0.182	0.182	0												
14	PGBAL009-19*Ch.plumosus* Montenegro	0.547	0.547	0.364	0.730	0.547	0.731	0.364	0.364	0.730	0.364	0.182	0.182	0	0											
15	PGCBG089-20*Ch.plumosus* Montenegro	0.364	0.364	0.182	0.547	0.364	0.547	0.182	0.182	0.547	0.182	0	0	0.182	0.182	0										
16	BSCHI661-17*Ch.plumosus* Poland	0.547	0.547	0.364	0.730	0.547	0.731	0.731	0.364	0.730	0.364	0.547	0.547	0.731	0.731	0.547	0									
17	BSCHI063-11*Ch.plumosus* Sweden	0.731	0.731	0.547	0.914	0.731	0.916	0.916	0.547	0.914	0.547	0.731	0.731	0.916	0.916	0.731	0.182	0								
18	BSCHI115-17*Ch.plumosus* Sweden	0.364	0.364	0.182	0.547	0.364	0.547	0.547	0.182	0.547	0.182	0.364	0.364	0.547	0.547	0.364	0.547	0.731	0							
19	BSCHI219-17*Ch.plumosus* Sweden	0.182	0.182	0	0.364	0.182	0.364	0.364	0	0.364	0	0.182	0.182	0.364	0.364	0.182	0.364	0.547	0.182	0						
20	BSCHI284-17*Ch.plumosus* Sweden	0.182	0.182	0	0.364	0.182	0.364	0.364	0	0.364	0	0.182	0.182	0.364	0.364	0.182	0.364	0.547	0.182	0	0					
21	BSCHI350-17*Ch.plumosus* Sweden	0.364	0.364	0.182	0.547	0.364	0.547	0.547	0.182	0.547	0.182	0.364	0.364	0.547	0.547	0.364	0.547	0.731	0	0.182	0.182	0				
22	BSCHI517-17*Ch.plumosus* Sweden	0.547	0.547	0.364	0.730	0.547	0.731	0.731	0.364	0.730	0.364	0.547	0.547	0.731	0.731	0.547	0	0.182	0.547	0.364	0.364	0.547	0			
23	BSCHI644-17*Ch.plumosus* Sweden	0.182	0.182	0	0.364	0.182	0.364	0.364	0	0.364	0	0.182	0.182	0.364	0.364	0.182	0.364	0.547	0.182	0	0	0.182	0.364	0		
24	GBDP44143-19*Ch.plumosus* UK	0.547	0.547	0.364	0.730	0.547	0.731	0.731	0.364	0.730	0.364	0.547	0.547	0.731	0.731	0.547	0	0.182	0.547	0.364	0.364	0.547	0	0.364	0	
25	GBDP44180-19*Ch.plumosus* Iran	2.028	2.406	2.217	2.592	2.406	2.595	2.217	2.217	2.219	2.217	2.028	2.028	2.217	2.217	2.028	2.217	2.406	2.406	2.217	2.217	2.406	2.217	2.217	2.217	0

We conducted the estimation of phylogenetic relationships in BEAST V1.10.4 (Suchard et al. 2018) by the Bayesian Markov-chain Monte-Carlo (MCMC) method, using the HKY+G substitution model as selected in MEGA 6. The determination of the appropriate model in MEGA 6 (Tamura et al. 2013) was performed. The strict clock as a clock model and the Yule process as a speciation model were used. We run MCMC for 10.000.000 iterations and 1000 iterations of burn in. Our analysis involved 61 nucleotide sequences, and we eliminated all positions with less than 95% site coverage. There were 579 positions in the final data set. We used the COI sequence of *Pagastiellaorophila* (Genbank accession number JN265047.1) as an outgroup.

**Table 2.** Estimates of evolutionary divergence between sequences of Nearctic *Ch.entis*-*Ch.plumosus* cluster. The number of base substitutions per site (%) from between sequences are shown. Analyses were conducted using the Kimura 2-parameter model (Kimura 1980).

№	Sequences	1	2	3	4	5	6	7	8	9	10	11	12	13
1	GBDPC429-14*Ch.entis* US, Michigan	0												
2	KF278213.1*Ch.entis* Canada, Quebec	0.914	0											
3	KF278212.1*Ch.entis* Canada, Quebec	1.099	0.182	0										
4	MGOCF102-16*Ch.entis* US, New York	0.914	0	0.182	0									
5	KJ085531.1*Ch.entis* Canada, Ontario	0.547	0.730	0.914	0.730	0								
6	KJ087284.1*Ch.entis* Canada, Ontario	0.547	0.730	0.914	0.730	0	0							
7	KJ089893.1*Ch.entis* Canada, Ontario	0.547	0.730	0.914	0.730	0	0	0						
8	GBDPC430-14*Ch.plumosus* US, Michigan	0.730	0.730	0.913	0.730	0.547	0.547	0.547	0					
9	SDP408034-15*Ch.plumosus* US, Minnisota	0.730	0.730	0.913	0.730	0.547	0.547	0.547	0	0				
10	GBDPC133-14*Ch.plumosus* Canada	1.285	0.364	0.547	0.364	1.099	1.099	1.099	1.098	1.098	0			
11	GBDPC138-14*Ch.plumosus* Canada	1.285	0.364	0.547	0.364	1.099	1.099	1.099	1.098	1.098	0	0		
12	GBDPC144-14*Ch.plumosus* Canada	0.730	0.730	0.913	0.730	0.547	0.547	0.547	0	0	1.098	1.098	0	
13	GBDPC166-14*Ch.plumosus* Canada	1.099	0.547	0.731	0.547	0.914	0.914	0.914	1.098	1.098	0.916	0.916	1.098	0

We also tried to get average estimates of divergence time between different branches and clusters that appear on the obtained phylogenetic tree (Figs 3, 4). The age of the most recent common ancestors (TMRCAs) for DNA clades was estimated in BEAST V1.10.4 (Suchard et al. 2018) by the MCMC method, using the HKY+G substitution model as selected in MEGA 6. We used a strict clock as a clock model and a constant size as a coalescent model, with the same calibration point assumed by Cranston et al. (2012). The time estimate of 36 million years ago (Mya) for the root node of the divergence between *Pagastiellaorophila* and all *Chironomus* species was used as a calibration point. We ran MCMC for 10.000.000 iterations and 1000 iterations of burn in. Tracer v1.7.1 was used to examine the BEAST log file and ESSs for each parameter, which were all > 200.

**Table 3.** Estimates of evolutionary divergence between sequences of Far Eastern *Ch.borokensis*-*Ch.suwai* cluster. The number of base substitutions per site (%) from between sequences are shown. Analyses were conducted using the Kimura 2-parameter model (Kimura 1980).

№	Sequences	1	2	3	4	5	6	7	8	9	10	11	12	13	14
1	AB740261.1*Ch.borokensis* Russia	0													
2	GBDP17582-15*Ch.plumosus* Japan	1.283	0												
3	GBDP17583-15*Ch.plumosus* Japan	1.468	0.547	0											
4	JCDB364-15*Ch.plumosus* Japan	1.469	0.182	0.364	0										
5	JCDB363-15*Ch.plumosus* Japan	1.469	0.182	0.364	0.000	0									
6	GBDP11685-12*Ch.plumosus* South Korea	0.364	1.654	1.839	1.841	1.841	0								
7	GBDP11686-12*Ch.plumosus* South Korea	0.182	1.469	1.654	1.656	1.656	0.182	0							
8	GBDP11687-12*Ch.plumosus* South Korea	0.182	1.469	1.654	1.656	1.656	0.182	0	0						
9	GBDP12282-12*Ch.plumosus* South Korea	0	1.283	1.468	1.469	1.469	0.364	0.182	0.182	0					
10	XJDQD1039-18*Ch.plumosus* China	1.468	0.913	1.467	1.097	1.097	1.839	1.654	1.654	1.468	0				
11	XJDQD1037-18*Ch.plumosus* China	1.468	0.913	1.467	1.097	1.097	1.839	1.654	1.654	1.468	0	0			
12	XJDQD1038-18*Ch.plumosus* China	1.468	0.913	1.467	1.097	1.097	1.839	1.654	1.654	1.468	0	0	0		
13	XJDQD1036-18*Ch.plumosus* China	1.468	0.913	1.467	1.097	1.097	1.839	1.654	1.654	1.468	0	0	0	0	
14	MN750315.1*Ch.plumosus* China	1.845	2.028	2.592	2.217	2.217	2.219	2.034	2.034	1.845	2.214	2.214	2.214	2.214	0

**Table 4.** Estimates of evolutionary divergence between sequences of *Ch.plumosus* from Finland and sequences of *Ch.balatonicus*, *Ch.muratensis* and Chironomussp.propeagilis. The number of base substitutions per site (%) from between sequences are shown. Analyses were conducted using the Kimura 2-parameter model (Kimura 1980).

№	Sequences	1	2	3	4	5	6	7
1	CHIFI299-16*Ch.plumosus* Finland, Satakunta	0						
2	CHIFI298-16*Ch.plumosus* Finland, Satakunta	1.099	0					
3	LEFIJ3947-16*Ch.plumosus* Finland, Regio aboensis	3.939	3.555	0				
4	LEFIJ3948-16*Ch.plumosus* Finland, Regio aboensis	3.566	3.573	3.362	0			
5	AF192190.1Chironomussp.propeagilis Russia	6.306	6.315	8.357	6.930	0		
6	JN016826.1*Ch.balatonicus* Russia, Saratov_terr.	3.372	3.378	3.555	0.547	6.315	0	
7	AF192194.1*Ch.muratensis* Russia	4.119	4.123	3.156	4.123	8.115	3.929	0

Recent research demonstrates that the range of divergence rates of the COI gene sequence in insects varies from 1.5% to 2.3% per 1 Mya (Jamnongluk et al. 2003; Stevens et al. 2006 etc.). In the study of tenebrionid beetles, Papadopoulou et al. (2010) obtained a divergence rate of 3.54% per 1 Mya for the COI gene (2.69% when combined with the 16S rRNA gene) under the preferred partitioning scheme and substitution model selected using Bayes factors. In our study, we used for calculations of divergence time these three commonly assumed mutation rates: 1.5%, 2.3%, and 3.54%. We calculated TMRCAs for the nodes 1–9 of the phylogenetic tree (Fig. 3). The obtained values are given in Table 5.

**Table 5.** Substitutions that distinguish *Ch.bonus* sequence from sequences in the Palearctic *Ch.plumosus* cluster; nonsyn. and syn. - nonsynonymous and synonymous substitutions respectively.

№	Substitution type	Position in the sequence	Codon	Position in codon	Ch.bonus sequence
1	nonsyn.	2	1	1^st^	MZ014021.1
2	syn.	212	71	1^st^	MZ014021.1
3	syn.	340	113	3^rd^	MZ014021.1 MZ014022.1 MZ014023.1
4	nonsyn.	609	203	2^nd^	MZ014023.1
5	nonsyn.	642	214	2^nd^	MZ014023.1
6	nonsyn.	644	215	1^st^	MZ014023.1

## ﻿Results

Based on morphological and chromosomal characters, we identified the larvae belonging to the genus *Chironomus* at the studied site as *Ch.bonus*. The morphology of *Ch.bonus* larvae from the South Caucasus is similar to that previously described for this species by Kiknadze et al. (1991b).

### ﻿Karyotype of *Ch.bonus* from the South Caucasus

**Figure 2.**
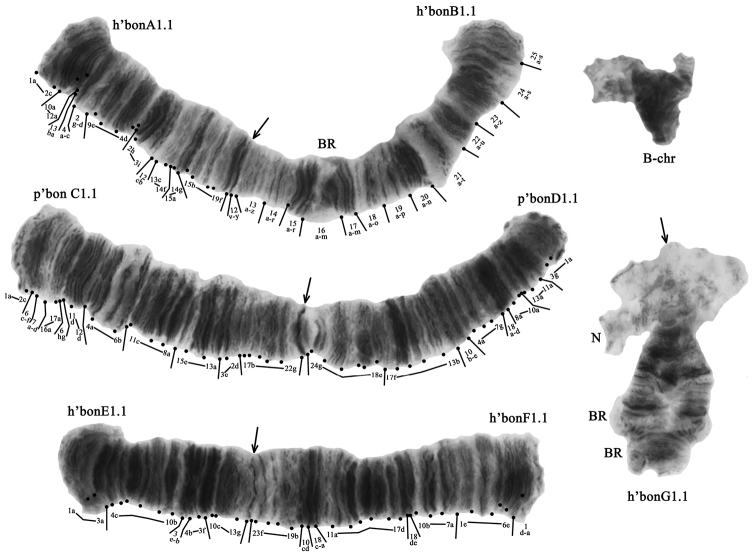
Karyotype of *Ch.bonus* from the South Caucasus; h’bonA1.1, h’bonB1.1 etc. – genotypic combinations of banding sequences; BR – Balbiani rings, N – nucleolus. Arrows indicate centromeric bands.

The diploid number of chromosomes in the *Ch.bonus* karyotype is 2n = 8 plus the B-chromosome. Such a picture for the *C.bonus* karyotype is based on the almost constant presence of an additional B-chromosome in the karyotype of each larva. The chromosome arm combinations are AB, CD, EF, and G (the “thummi” cytocomplex) (Fig. 2). The chromosomes AB and CD are metacentric, EF is submetacentric, and G is telocentric. Arm G homologues are paired in the nucleolus and Balbiani rings (BRs) regions. The centromeric bands are easily identifiable. There is one nucleolus and two BRs on the arm G, and one BR is present on the arm B.

### ﻿Banding sequences and chromosomal polymorphism of *Ch.bonus* from the South Caucasus

The karyotype of *Ch.bonus* from the South Caucasus is monomorphic. The banding sequences of all the chromosome arms of *Ch.bonus* are identical to those of *Ch.plumosus*. The difference between the karyotypes of both species is the presence of one additional B-chromosome in almost all studied *Ch.bonus* larvae. In total, there are 7 banding sequences in the *Ch.bonus* banding sequences pool (Fig. 2):

h’bonA1 1a-2c 10a-12a 13ba 4a-c 2g-d 9e-4d 2h-3i 12cb 13c-14f 15a-14g 15b-19f C*

h’bonB1 25s-a 24s-a 23z-a 22u-a 21t-a 20n-a 19p-a 18o-a 17m-a 16m-a 15r-a 14r-a 13z-a 12y-v C**

p’bonC1 1a-2c 6c-f 7a-d 16a-17a 6hg 11d-12d 4a-6b 11c-8a 15e-13a 3c-2d 17b-22g C

p’bonD1 1a-3g 11a-13a 10a-8a 18d-a 7g-4a 10e-b 13b-17f 18e-24g C

h’bonE1 1a-3e 5a-10b 4h-3f 10c-13g C***

h’bonE1 1a-3a 4c-10b 3e-b 4b-3f 10c-13g C*

h’bonF1 1a-d 6e-1e 7a-10b 18ed 17d-11a 18a-c 10dc 19a-23f C

h’bonG1 not mapped

* revised mapping by Golygina and Kiknadze (2008, 2012)

** mapped according to system of Maximova-Shobanov (Maximova 1976; Shobanov 1994), mapping revised by Golygina and Kiknadze (2008).

*** mapped according to Keyl (1962).

### ﻿Results of phylogenetic analysis of COI gene sequences of *Ch.bonus* and estimated ages of the most recent common ancestors (TMRCAs) for DNA clades

Overall, we successfully obtained three complete COI gene sequences of *Ch.bonus* from six larvae from the South Caucasus. (MZ014021.1: 627 bp, base composition is 25.99% A, 36.84% T, 16.91% G, and 20.26% C; MZ014022.1: 658 bp, base composition is 26.59% A, 36.17% T, 16.57% G, and 20.67% C; MZ014023.1: 650 bp, base composition is 27.08% A, 35.38% T, 16.77% G, and 20.77% C). Each of the three sequences had a different haplotype. This is the first DNA data obtained for *Ch.bonus*.

**Figure 3.**
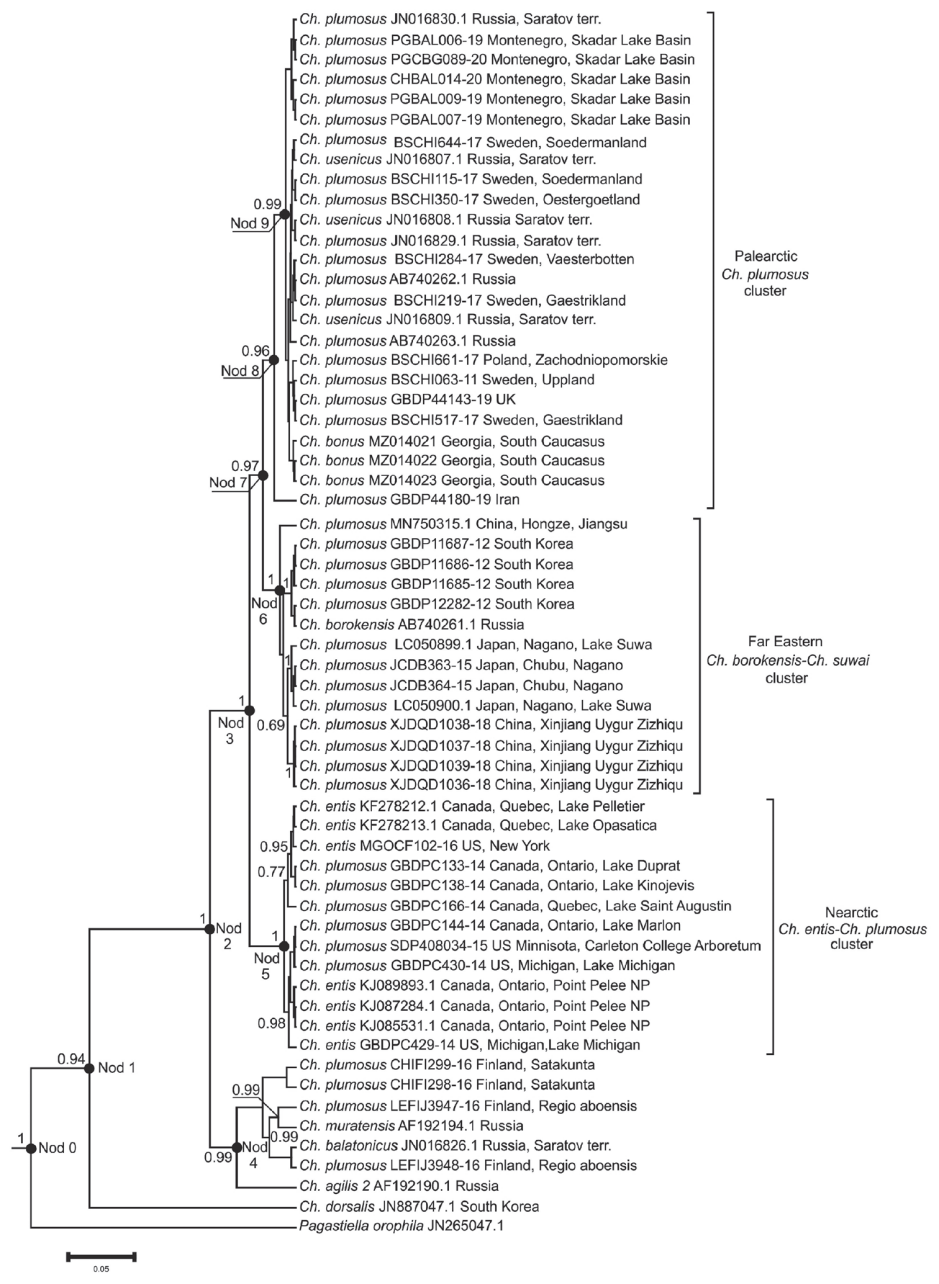
Phylogenetic tree of *Chironomus* species estimated by the Bayesian inference (BA). Support values are given if they exceed 0.5. The numbers at the nodes indicate posterior probabilities; Node 1, Node 2 etc. – nodes of the tree for which TMRCAs were calculated.

The resulting phylogenetic tree (Fig. 3) represents a very complex pattern, with several obvious clusters with rather high probabilities. We conditionally named them the Palearctic *Ch.plumosus* cluster, the Far Eastern *Ch.borokensis*-*Ch.suwai* cluster, and the Nearctic *Ch.entis*-*Ch.plumosus* cluster.

**Figure 4.**
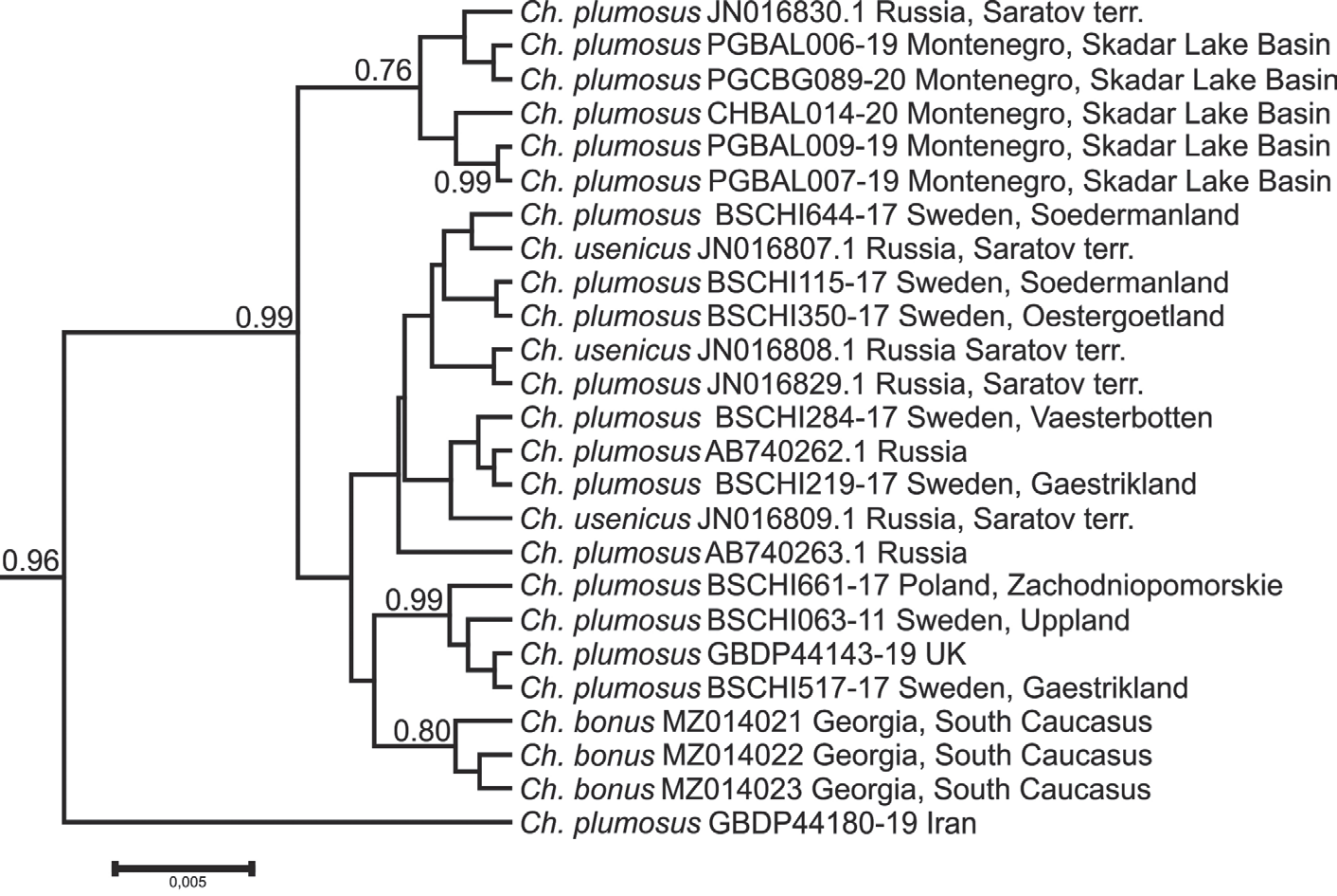
Phylogenetic tree of Palearctic *Ch.plumosus* cluster estimated by the Bayesian inference (BA). Support values are given if they exceed 0.5. The numbers at the nodes indicate posterior probabilities.

The Palearctic *Ch.plumosus* cluster (Figs 3, 4), is formed mostly by *Ch.plumosus* sequences from Western and Eastern Europe (UK, Sweden, Poland, Montenegro, and the European part of Russia). The only available sequences of *Ch.usenicus* from Russia (Saratov Terr.) and, surprisingly, sequences of *Ch.bonus* obtained in this study, are also included in this cluster. It is formed by sequences obtained from material identified through both karyological and morphological analyses (all *Ch.usenicus* and *Ch.bonus* sequences, together with a few *Ch.plumosus* ones, i.e., JN016830.1, JN016829.1, AB740262.1, AB740263.1), and we therefore named it the Palearctic *Ch.plumosus* cluster.

The Far Eastern *Ch.borokensis*-*Ch.suwai* cluster mostly formed by *Ch.plumosus* sequences from the Far East (China, South Korea, and Japan) and a sequence of *Ch.borokensis* from Russia. We named this branch as the Far Eastern *Ch.borokensis*-*Ch.suwai* cluster because this particular *Ch.borokensis* sequence (AB740261.1) was obtained from the material identified through karyological analysis (Kondo et al. 2016). According to the BOLD database, *Ch.plumosus* sequences from the Far East were obtained from specimens identified only through morphological analysis. Perhaps the observed picture is an error in species identification, which can happen quite often when chromosomal analysis is not involved, and at least some of these *Ch.plumosus* specimens from the Far East could actually be *Ch.borokensis*. On the other hand, at least two Japanese sequences that we used in our study from Lake Suwa could be *Ch.suwai*. We assume this because Lake Suwa is the type locality for the species. According to Golygina et al. (2003), the karyotype of *Ch.suwai* is closely related to that of *Ch.borokensis* as indicated by many common banding patterns, but it differs by the much smaller size of the centromeric bands. Also, Golygina et al. (2003) suppose that the true *Ch.plumosus* does not occur in Japan.

Almost the same pattern is observed in the Nearctic *Ch.entis*-*Ch.plumosus* cluster, consisting equally of *Ch.plumosus* and *Ch.entis* sequences. According to the data from Proulx et al. (2013), just two sequences of *Ch.entis* (KF278213.1 and KF278212.1) and three sequences of *Ch.plumosus* (GBDPC133-14/KF278209.1, GBDPC138-14/KF278210.1 and GBDPC144-14/KF278216.1) from this cluster were obtained from material identified through karyological analysis. Except for these sequences, it is most likely an error in species identification as well, and at least some of the *Ch.plumosus* sequences presented in BOLD from Northern America could actually be *Ch.entis* and vice versa. Also, in this cluster, there are no *Ch.plumosus* or *Ch.entis* sequences from the Palearctic region. Given all this data, we named this cluster the Nearctic *Ch.entis*-*Ch.plumosus* cluster.

In addition to the above-mentioned obvious clusters of the tree (Fig. 3), there is a fourth ambiguous cluster formed by *Ch.balatonicus*, *Ch.muratensis*, Chironomussp.propeagilis, and all *Ch.plumosus* sequences from Finland. We know from the BOLD database that these *Ch.plumosus* sequences were obtained from adults identified just through morphological analysis. Perhaps this pattern is an error in species identification, and these Finnish specimens could actually belong to other already known or even previously undescribed species.

### ﻿Genetic distances

Calculated pairwise sequence distances (Tables 1–4) consisting of the estimated number of base substitutions per site using the K2P model (Kimura 1980) show an interesting pattern. Proulx et al. (2013), who used genetic, morphological, and karyological information to discriminate *Chironomus* species from Canada, showed that intraspecific K2P distances for *Chironomus* species characterized by the COI gene ranged from zero to 3%. These values also could be used as a reference for distinguishing *Chironomus* species in the present work, but data on the COI gene should be complemented with other methods. In our study, the distances between the Palearctic *Ch.plumosus* cluster’s sequences are less than 3% and range from 0 to 2.595% (Table 1). If data on the Iranian *Ch.plumosus* are removed, the distances between all other sequences in this cluster are less than 1%, ranging from 0 to 0.914%. Distances between the sequences of *Ch.bonus* obtained in this study are very small, varying from 0.182% to 0.364%. The sequences of *Ch.usenicus* and of several individuals of *Ch.plumosus* from Russia (Saratov Terr.), Sweden, and Montenegro are closest to those of *Ch.bonus* in terms of distances.

Almost the same pattern is observed in the Nearctic *Ch.entis*-*Ch.plumosus* cluster, where the distances between the sequences are also lower than the 3% range, varying from 0 to 1.285% (Table 2).

In the Far Eastern *Ch.borokensis*-*Ch.suwai* cluster, the distances between the sequences are also lower than the 3% range, varying from 0 to 2.217% (Table 3). If we disregard *Ch.plumosus* sequence MN750315.1 from China, Hongze, Jiangsu, the distances between all other sequences in this cluster are significantly less, reaching only 1.839%.

At the same time, the average distances between the various clusters exceed the 3% threshold. The distance between Palearctic *Ch.plumosus* and Far Eastern *Ch.borokensis*-*Ch.suwai* clusters is 3.55%. The distance between Palearctic *Ch.plumosus* and Nearctic *Ch.entis*-*Ch.plumosus* clusters is 3.75%. Finally, the distance between Far Eastern *Ch.borokensis*-*Ch.suwai* and Nearctic *Ch.entis*-*Ch.plumosus* clusters is 5.98%.

In the fourth cluster, which contains *Ch.plumosus* sequences from Finland, the distances between the sequences are generally higher than the 3% range (Table 4). Interestingly, the distances between the sequence of Chironomussp.propeagilis and all other sequences are pretty high, varying from 4.123 to 8.357%. On the other hand, analogous distances in the case of *Ch.muratensis* are also fairly high, varying from 4.119 to 8.115%. However, the distances between the sequence of *Ch.balatonicus* and most of the Finnish sequences of *Ch.plumosus* are also high enough, varying from 3.372 to 3.555%. At the same time, the distance between the sequence of *Ch.balatonicus* and one Finnish sequence of *Ch.plumosus* from Regio aboensis (LEFIJ3948-16) is just 0.547%, which is much lower than the 3% range, and we therefore can assume that this *Ch.plumosus* sequence could actually belong to *Ch.balatonicus*. Moreover, the distances between the three other *Ch.plumosus* sequences from Finland, i.e., that from Regio aboensis (LEFIJ3947-16) and two from Satakunta (CHIFI299-16 and CHIFI298-16), are relatively high, varying from 3.555 to 3.939%. At the same time, the distance between the two latter sequences is just 1.099%, which is lower than the 3% threshold. Considering the tree topology (Fig. 3) and genetic distances between the sequences, we can suggest that a particular sequence from Regio aboensis, on the one hand, and another two sequences from Satakunta, on the other hand, belong to two different, possibly previously undescribed species. This assumption is quite possible because Michailova (2001) found in Finland (Lake Arima and Lokka Reservoir) two unknown karyotypes similar to those of the *Ch.plumosus* group. She proposed that at least one of these karyotypes could correspond to *Ch.coaetaneus* Hirvenoja, 1998 (Hirvenoja 1998), which may be related to *Ch.plumosus*.

Some sequences in the Palearctic *Ch.plumosus* cluster initially were not complete, and it was hard to make a good comparison. But still, we found a small number of substitutions that distinguish the sequences of *Ch.bonus* from other sequences in the Palearctic *Ch.plumosus* cluster. Overall, we found six substitutions of that kind (Table 5). Four of them are nonsynonymous substitutions, and the remaining two are synonymous ones. Among them, there is a single unique 340-position substitution that was found in all three sequences of *Ch.bonus*. All other substitutions are also found in certain sequences from other clusters. Only this unique substitution clearly distinguishes *Ch.bonus* from other species in our entire data set.

### ﻿Tempo of diversification

According to the obtained results, the earliest split of the *Ch.plumosus* group of sibling species occurred during the Late Miocene (7,3–5,3 Mya) and early Pliocene (5,3–2,58 Mya) epoch (Fig. 3; Table 6, node 2), dating back to 5.75–3.43 Mya (substitution rates for all earliest and latest estimates in this chapter are 1.5% and 3.54%, respectively). The most recent common ancestor of all Palearctic *Ch.plumosus*, Far Eastern *Ch.borokensis*-*Ch.suwai*, and Nearctic *Ch.entis*-*Ch.plumosus* clusters lived 2.88–1.72 Mya (Fig. 3; Table 6, node 3). This split occurred in the Early Pleistocene. The most recent common ancestor of all members of the Nearctic *Ch.entis*-*Ch.plumosus* cluster lived 0.638–0.378 Mya (Fig. 3; Table 6, node 5). The split between Palearctic *Ch.plumosus* and Far Eastern *Ch.borokensis*-*Ch.suwai* clusters dates back to 1.97–1.17 Mya (Fig. 3; Table 6, node 7). The most recent common ancestor of all members of the Far Eastern *Ch.borokensis*-*Ch.suwai* cluster lived 0.906–0.539 Mya (Fig. 3; Table 6, node 6). The most recent common ancestor of all members of the Palearctic *Ch.plumosus* cluster lived 1.288–0.759 Mya (Fig. 3; Table 6, node 6). If we disregard the Iranian *Ch.plumosus* sequence, the most recent common ancestor of all other members of the Palearctic *Ch.plumosus* cluster dates back even later, 0.517–0.307 Mya (Fig. 3; Table 6, node 6).

**Table 6.** Estimations of the age of the most recent common ancestors (TMRCAs) for DNA clades.

Node number	Mean value (Mya)	Stdev.	95% HPD interval	ESS
Divergence rate 1.5%				
Node 0	29.177	5.499	19.403, 40.569	6368
Node 1	17.288	3.874	10.391, 25.051	5221
Node 2	5.746	1.293	3.321, 8.280	4724
Node 3	2.883	0.698	1.689, 4.333	4562
Node 4	3.895	0.995	2.147, 5.844	5323
Node 5	0.638	0.212	0.284, 1.057	3230
Node 6	0.906	0.26	0.447, 1.427	3866
Node 7	1.971	0.505	1.027, 2.927	4836
Node 8	1.288	0.395	0.612, 2.077	5470
Node 9	0.517	0.169	0.229, 0.852	3570
Divergence rate 2.3%				
Node 0	24.538	4.519	15.765, 33.035	7072
Node 1	13.716	2.992	8.518, 20.073	5990
Node 2	4.380	0.933	2.639, 6.220	5168
Node 3	2.204	0.511	1.288, 3.228	5429
Node 4	2.962	0.727	1.683, 4.467	5722
Node 5	0.481	0.155	0.217, 1.378	3759
Node 6	0.692	0.197	0.335, 1.071	4840
Node 7	1.503	0.375	0.820, 2.230	5210
Node 8	0.979	0.296	0.466, 1.570	5899
Node 9	0.395	0.129	0.177, 0.649	3576
Divergence rate 3.54%				
Node 0	21.017	3.923	13.900, 28.841	6753
Node 1	11.123	2.492	6.596, 16.182	5914
Node 2	3.431	0.763	2.043, 4.944	4731
Node 3	1.715	0.410	1.013, 2.548	4676
Node 4	2.317	0.586	1.257, 3.468	5414
Node 5	0.378	0.124	0.164, 0.624	3784
Node 6	0.539	0.151	0.271, 0.835	4660
Node 7	1.170	0.295	0.634, 1.743	4902
Node 8	0.759	0.229	0.349, 1.206	6108
Node 9	0.307	0.101	0.137, 0.509	3832

## ﻿Discussion

Studied larvae of *Ch.bonus* have a monomorphic karyotype, with its details similar to those previously described for this species by Kiknadze et al. (1991a). Following Proulx et al. (2013), we can conclude that the genetic distances between observed Palearctic *Ch.plumosus*, Far Eastern *Ch.borokensis*-*Ch.suwai*, and Nearctic *Ch.entis*-*Ch.plumosus* clusters exceed the 3% range. This result leads us to some interesting conclusions about the level of divergence between the Palearctic and Nearctic populations of *Ch.plumosus*. Our calculations show that the distance (3.75%) between the Palearctic and Nearctic sequences of *Ch.plumosus* exceeds the 3.0% range for *Chironomus* species. One can say that since the divergence time of 2.88–1.72 Mya (Fig. 3; Table 6, node 3), the Nearctic populations of *Ch.plumosus* have already become a separate species.

We can propose two possible explanations for the observed pattern within the Palearctic *Ch.plumosus* cluster (Fig. 4), which also included the *Ch.bonus* sequences obtained during this study. The first explanation is similar to that earlier suggested by Guryev and Blinov (2002), who found that populations of *Ch.entis* and *Ch.plumosus* did not group together on the trees based on the mitochondrial cytb gene according to their species affiliation. They suggested that it could result from interspecific hybridization followed by recurrent crosses. Consequently, the offspring inherited mtDNA of one of the parental species. In this case, even an insignificant selective advantage of this mtDNA is able to lead to the rapid fixation of the new haplotype in the population. Later, Polukonova et al. (2009) in the work where they studied the COI sequences of cytologically identified *Ch.usenicus*, also inclined to this explanation when some *Ch.usenicus* and *Ch.plumosus* COI gene sequences were almost identical. In addition, Proulx et al. (2013) reported that the COI sequences of cytologically identified *Ch.plumosus* and *Ch.entis* larvae collected from Canada cluster together, and some of these sequences are identical. We can therefore assume that the separation of *Ch.bonus* and *Ch.plumosus* from a common ancestor could occur long ago. During this time, in the gene pool of *Ch.bonus*, a unique, separate line of COI gene emerged, but then, an interspecific hybridization between a male of *Ch.bonus* and a female of *Ch.plumosus* occurred. In the hybrid offspring, the *Ch.plumosus* COI sequence gradually replaced that of *Ch.bonus*.

The observed pattern also can be explained by a relatively recent separation of the two species, with *Ch.plumosus* being a parental species to *Ch.bonus*. The COI gene sequences of these species are therefore very similar, with a very low number of new substitutions in the *Ch.bonus* lineage. However, we discovered a number of substitutions that clearly distinguish *Ch.bonus* from *Ch.usenicus* and *Ch.plumosus* from European populations (Table 5).

We can assume that the *Ch.plumosus* group originated from the common ancestor during the Pliocene of 5.75–3.43 Mya. However, since we have certain DNA data only for seven species of the *Ch.plumosus* group out of 14, this temporary estimate could change in the future in favor of the older age. At the same time, the obtained age of the most recent common ancestor of the *Ch.plumosus* group corresponds rather well to the estimations by Demin and Polukonova (2008) (5.8–3.7 Mya), despite the substantially lower amount of data available for those authors.

We can be more confident about the age of the most recent common ancestors of species constituting the Palearctic *Ch.plumosus*, Far Eastern *Ch.borokensis*-*Ch.suwai*, and Nearctic *Ch.entis*-*Ch.plumosus* clusters. It is possible that the age of the Palearctic *Ch.plumosus*, Far Eastern *Ch.borokensis*-*Ch.suwai*, and Nearctic *Ch.entis*-*Ch.plumosus* clusters is 0.638–0.378, 0.906–0.539 and 1.288–0.759 million years (Myr) respectively. The age of European populations of *Ch.plumosus* is approximately 0.517–0.307 Myr. We therefore suggest that observed clusters have arisen relatively recently in the Middle Pleistocene sub-epoch.

We concluded that the most recent common ancestor of the *Ch.plumosus* group originated in the Pliocene epoch (5.3–2.58 Mya). It is known that this epoch is characterized by the appearance of a new type of biome, the first true grasslands, due to the retreat of the forests associated with the gradual cooling of the climate that began in the previous epochs. True grasslands and Serengeti-like communities of grazing animals probably did not appear until the Late Miocene in the New World and the Pliocene in the Old World (ca. 5 Mya) (Pärtel 2005).

Due to the heterogeneity of the landscapes, new stagnant water bodies became increasingly abundant. In contrast to lowland rivers, which usually have similar environmental parameters, each of these stagnant water bodies was often characterized by a unique combination of size, shape, depth, temperature profile, mineralization level etc. This variation in environmental parameters could easily lead to differences in breeding time between various populations or individuals that can potentially lead to reproductive isolation and the emergence of new species. We suggest that the species divergence in this group could have been caused by invasion of their common ancestor into newly originated water bodies.

## ﻿Data availability statement

The data (Figs and Tables) that support this study are available in FigShare at https://doi.org/10.6084/m9.figshare.17060912.v1 and https://doi.org/10.6084/m9.figshare.17060666.v1.
